# Preceptorship during health personnel students’ clinical studies in nursing homes—An institutional perspective on an intervention

**DOI:** 10.1002/nop2.197

**Published:** 2018-09-14

**Authors:** Bjørg Aglen, Vera Louise Sørø, Arne Orvik, Gørill Haugan

**Affiliations:** ^1^ Department of Public Health and Nursing, Faculty of Medicine and Health Sciences Norwegian University of Technology and Science Trondheim Norway; ^2^ Department of Health Sciences, Faculty of Medicine and Health Sciences Norwegian University of Technology and Science Ålesund Norway

**Keywords:** clinical studies, institutional theory, intervention, nursing home, organizational change, preceptorship, qualitative study

## Abstract

**Aim:**

The recruitment and retention of preceptors to mentor health professional students and apprentices in their clinical studies are not easy. The aim of this study was to investigate factors that hindered the implementation of an intervention intended to improve the working conditions for preceptors in nursing homes. The preceptorship was related to clinical studies for health professional students and apprentices.

**Design:**

A qualitative, explorative design was applied.

**Methods:**

Qualitative data were collected in September 2014 by means of focus groups with preceptors and key informant interviews. The data were prepared by thematic analysis and interpreted in the light of institutional theory.

**Results:**

The intervention to improve the working conditions for preceptors was hampered by institutional traits involving rule‐like perceptions of “want to,” “ought to” and “have to.” Precepting was preserved as an individual task of the preceptors and was not considered a daily activity in nursing homes.

**Conclusions:**

To improve the working conditions for preceptors in nursing homes and thus improve recruitment and retention among preceptors, the nursing home leaders should address institutional traits related to preceptorship.

## 1 INTRODUCTION

Clinical studies in healthcare settings represent 50% of the academic credits in the education of a Registered Nurse (RN) in Europe (Lahtinen, Leino‐Kilpi, & Salminen, 2014). Thus, clinical studies of high quality are crucial to reaching learning outcomes. Accordingly, a high standard of preceptorship in students’ clinical studies is required. The healthcare service itself underlines the importance of clinical studies during which students are socialized into professional standards to facilitate a smooth transition into work after graduation (Mallaber & Turner, 2006). A positive learning environment and high‐quality preceptorship are ranked as the most important factors for learning and thriving by health professional students (Jokelainen, Turunen, Tossavainen, Jamookeeah, & Coco, 2011; Levett‐Jones, Lathlean, Higgins, & McMillan, 2009). Experiences of an including and supportive learning environment motivate to future recruitment in nursing homes (NHs) after graduation (Algoso, Peters, Ramjan, & East, 2015; Lea, Mason, Eccleston, & Robinson, 2016). Consequently, NH leaders should prioritize and facilitate preceptorship and a positive learning environment as part of their daily work. However, preceptors across different occupational backgrounds report a lack of competence, time for preparation, recognition and organizational support as barriers to effective supervision as well as barriers to retain in the role as a preceptor (Caspersen & Kårstein, 2013; Forber, DiGiacomo, Davidson, Carter, & Jackson, 2015; Omansky, 2010; Trede, Sutton, & Bernoth, 2016). Studies focusing on nursing students’ clinical studies indicate an urgent need for coordination and leadership about preceptors’ working conditions (Forber et al., 2015; O'Driscoll, Allan, & Smith, 2010; Trede et al., 2016). This statement refers to the estimated shortage of workforce in the future combined with increased demands of the healthcare services (Forber et al., 2015). Accordingly, efforts to attract and retain essential competence (RNs) in the healthcare services are crucial, especially in the primary healthcare sector. It has been evident for at least the last decade that experiences from clinical studies predict nurses’ future choice of workplace (Algoso et al., 2015; Forber et al., 2015; Lea et al., 2016; Levett‐Jones et al., 2009). While previous research has viewed preceptorship as an individual rather than a collective responsibility, an overall need calls for studies explaining preceptorship at an organizational level (Trede et al., 2016).

## 2 BACKGROUND

The concepts of preceptorship and mentorship are used interchangeably (Budgen & Gamroth, 2008; Yonge, Billay, Myrick, & Luhanga, 2007). Nevertheless, preceptorship and mentorship are concepts referring to different practices and roles. The preceptor role encompasses facilitating students’ socialization into the professional role and accessing learning situations, and often includes assessment of the students’ abilities to become clinical practitioners or a member of the staff. Thus, the preceptor is supposed to be a competent practitioner who facilitates students’ learning and socialization into the clinical work at the actual workplace (Yonge et al., 2007). Neither the student nor the preceptor chose each other; instead, they are formally pared according to the formal educational programme for clinical practice negotiated between the place of education and the place of practice. In contrast, the mentee and the mentor voluntary select each other, the mentee because of the mentors’ competencies and experiences within a certain domain of knowledge and the mentor because of an interest in guiding the actual mentee in his learning trajectory, independent of specific clinical workplaces (Yonge et al., 2007). Thus, this relation is more personal and less framed of formalities than the preceptor–preceptee relation is. The purpose of the mentee–mentor relation is growth, both personal and professional, and the relation does not include any formal obligations to evaluate or assess the mentees’ competence for transition into a specific workforce. Due to the distinction between the concepts of preceptorship and mentorship, the authors find preceptorship the most appropriate term for the relationship between a clinician and a student or apprentice in this study. NH clinical staff usually consist of RNs, nursing associates and nurse assistants. The latter are personnel without formal healthcare education. In Norway, the education of a nursing associate encompasses 2 years of education in secondary school, followed by 2 years of apprenticeship in the healthcare services before gaining a formal authorization as a nursing associate. In this study, apprentices are students of associate nursing. Research related to placement and precepting for associate nurses is scarce.

Health professional education escalated from vocational training to higher level education during the 1970s (Forber et al., 2015; Laiho, 2010). This academization of health professionals’ education included further clinical studies as a practicum or internship. In Norway, the healthcare services funded by the government are obligated by law to facilitate learning in clinical studies for students and apprentices. However, each healthcare unit prioritizes their spending within their budget. The cooperation between the healthcare services and the health professional educators is not regulated by law but depends on cooperative agreements (Kårstein & Caspersen, 2014; Universitets‐ og høgskolerådet, 2016). However, completion of the agreed requirements takes place on an individual level, between the preceptors and lecturers, and not on an organizational level (Kårstein & Caspersen, 2014; Trede et al., 2016). The preceptorship model (Budgen & Gamroth, 2008) is supposed to bridge the knowledge–practice gap by enhancing the preceptors’ competence in observation and the educational curricula. This model is frequently used to facilitate students’ learning during clinical studies (Billay & Myrick, 2008).

Research has outlined preceptorship as the most important factor for a positive learning experience during clinical studies (Berntsen & Bjørk, 2010; Hecimovich & Volet, 2011; O'Driscoll et al., 2010; Trede et al., 2016). In the preceptorship model, one or two students are assigned to a preceptor, who is an health professional with clinical experience and employed in the actual unit (Budgen & Gamroth, 2008). Preceptorship quality largely depends on the preceptor's competence (Billay & Myrick, 2008). Hence, previous studies have focused on the student–preceptor relationship and the collaboration between the preceptor and the lecturer. This arrangement might have led to an underestimation of the workplace culture and leadership influence on the preceptors’ ability to fulfil their role (Trede et al., 2016). Many health professionals find the preceptor role to be interesting and stimulating, but some report a lack of dedicated time for preceptorship along with a heavy workload and lack of support from colleagues and leaders (Omansky, 2010). The tension between the role as a health professional and the role as an educator might lead to a certain unwillingness to take on the preceptor role (Caspersen & Kårstein, 2013; Trede et al., 2016). The influence of workplace cultures and learning environments on the preceptors’ role is rarely investigated in the context of health professional education (Trede et al., 2016). Sufficient available time to prepare and accomplish precepting, active follow‐up from leaders and acknowledgement of the preceptor role from colleagues should be provided (Hall, 2014; Jokelainen, 2013). Such arrangements may increase both recruitment and retention of preceptors.

To improve preceptors’ working conditions and consequently the students’ learning outcomes in NHs, we developed a pilot project including an intervention framed “interprofessional preceptor‐team.” This study investigates factors that hindered the implementation of this intervention, which provoked resistance among both the leaders and the preceptors. Since the leaders and the preceptors agreed in advance to act in line with the intervention, we did not foresee this resistance. Hence, a scrutiny of this resistance might provide valuable insight for future change plans and processes in NHs. From the perspective of institutional theory, the aim of this study was to investigate factors that hindered the implementation of the intervention.

## 3 THEORETICAL FRAMEWORK

Organizational change implies a comprehensive change in collective human behaviour, often prompted by resistance to change (Amis & Aïssaoui, 2013; Nilsen, 2015; Scott, 2014). The main challenge in quality improvement is not a lack of knowledge and interventions but a lack of successful implementation of interventions (Amis & Aïssaoui, 2013; Fixsen, Naoom, Blase, Friedman, & Wallace, 2005; Proctor et al., 2011). Implementation theory has been criticized for its tendency to view organizations as rational entities acting in compliance with formal, rational decisions to achieve clear, unambiguous goals (Nilsen, 2015), such as facilitating students’ and apprentices’ clinical studies to ensure future recruitment of health personnel staff. However, substantial empirical research has proved that organizations produce both rational and irrational practices (Scott, 2014), indicating that organizations act in both logical and illogical ways.

Institutional theory grasps the irrational sides of organizations. This theory acknowledges that organizations along with explicit formalized structures have implicit informal cultures guiding organizational behaviour. Institutional theory challenges the notion of organizations as instruments to achieve determinate goals. Instead, institutions are socially constructed organizations. Institutional theory highlights how cultural and social processes in a working collective develop patterns of the most preferred ways to think and act, which achieve a rule‐like status (Palthe, 2014). Institutions emerge when individuals in an organization interact over time and come to accept shared perceptions of reality; these perceptions create shared meanings and lead to repeated patterns of behaviour. These patterns of rule‐like status are conceptualized as institutional traits, that is, they form the institution (Scott, 2014) by means of three different kinds of traits: (a) cultural–cognitive, (b) normative and (c) regulative (Scott, 2014). The behavioural reasoning for the regulative traits is “have to,” for the cultural–cognitive, it is “want to,” and for the normative, it is “ought to” (Palthe, 2014). The “want to” represents traits of personal value that the recipients feel an intrinsic motivation to accomplish; this might be expressed as the following: “We want to precept students because we find it interesting and important.” However, the accomplishment of norms and regulative traits are more extrinsically motivated and might be expressed as: “We have to precept students because we are obligated by laws and regulations” and “we ought to precept students’ even if we really do not have the time for it.” These traits might interplay. For instance, the majority's “want to” tends to be the minority's “ought to” (Palthe, 2014).

Over time, these traits are taken for granted; the collective becomes familiarized with the traits, which then largely penetrate social activities and interactions. Newcomers are introduced and more or less prompted to accept “the way we do it here.” After some time, the traits are unconsciously and invisibly preserved and not discoverable until they are exposed to change. Confronted with change, those familiarized with the traits might feel provoked and refuse to change. The strength of resistance to change reflects the institutional forces. This spontaneous and immediate reaction of resistance represents a guide to the unconscious traits of behaviour and reasoning, embedded in the working collective. Therefore, it is important to investigate and analyse the resistance, both among those exposed to the change and among those planning to implement new ways of activities and interaction in the organization (Thelen & Mahoney, 2010). Both parts might then be able to make conscious choices about the efforts needed to change.

## 4 METHOD

In a qualitative, explorative design (Patton, 2015), a strategic sample of informants were recruited as participants. Data were collected by means of two focus group (FG) discussions and individual interviews of two key informants (Brinkmann & Kvale, 2015; Patton, 2015). An FG represents a discussion arena for informants to share and compare individual experiences on a certain theme (Barbour & Flick, 2007; Kitzinger, 1994; Orvik, Larun, Berland, & Ringsberg, 2013). Background information was gathered using a questionnaire including formal education and experiences as health professionals and as preceptors, frequencies of being preceptors and attending the preceptor‐team. Key informants (KIs) are informants who possess thorough and strategic information about the phenomenon of interest or play a key role in relation to the question under investigation (Patton, 2015). Thematic analysis was used to reveal patterns and themes about the phenomenon of interest (Braun & Clarke, 2006). Thematic analysis implies the integration of disparate pieces of data in a data set to constitute themes that reflect the meaning of the data. Thematic analysis emphasizes the context of the data material and is suitable for both manifest and latent analyses (Braun & Clarke, 2006).

### 4.1 Intervention, participants and setting

The present study is based on evaluation results of a pilot project to improve the working conditions for preceptors in a Norwegian NH (Kvam, 2015). This NH faced problems recruiting and retaining preceptors among RNs, nursing associates and physiotherapists (Kvam, 2015). The intention of the intervention was to increase the preceptors’ motivation (for working as preceptors) by improving their working conditions. An external advisor in cooperation with the NH's preceptors and leaders led the development of the intervention. The external advisor was an experienced RN holding a master's degree in management and employed at a governmentally funded agency to facilitate development in primary healthcare organizations. The intervention involved the establishment of a preceptor‐team and the facilitation of the preceptorship as an integrated part of the daily work by the NH leaders. The intention was to provide an arena for the preceptors to share experiences and mutual support. Sixteen healthcare workers participated in this pilot project: seven auxiliary nurses, six RNs, two physiotherapists and one social educator, altogether representing four wards. The precepting activities were planned by the four ward leaders in collaboration with the preceptors at their respective wards. The ward leaders were supposed to provide dedicated time for precepting students as well as time for the preceptors to attend the preceptor‐team. Furthermore, the preceptors were promised time to attend preparation courses at the educational sites ahead of each period of clinical studies. As Result section indicates, the implementation of the planned improvements did not turn out as expected. The pilot project implementing the intervention framed “interprofessional preceptor‐team” lasted a year followed by an evaluation (Kvam, 2015). In total, 15 preceptors participated in the interprofessional preceptor‐team, among who <10 attended five of the seven team meetings. Those not showing up did not inform the external advisor that coordinated the team meetings about their absence. The team participants claimed that collisions with other imposed meetings and high level of sick leave at the wards caused their absence; they said that they could not leave the ward to attend the team. The participants were supposed to use the email system to ask their leaders for leeway for both precepting and for attending the team meetings. The leaders reported that preceptors at one of four wards had done so. The others had not asked their leaders for leeway. The external advisor concluded that the intervention did not work out as intended and asked the research team to investigate the reasons why. Hence, this study evaluates factors hindering the implementation of the interprofessional preceptor‐team and provides needed insight about the feasibility of the present intervention for implementation in other NHs.

One NH comprising of four wards signified the study setting. At this NH, students in nursing, physiotherapy, social education as well as nursing associate apprentices conducted their clinical studies. The NH personnel were RNs, nursing associates and nursing assistants. One ward also employed physiotherapists. All preceptor‐team members were invited to participate in forming two FGs representing four wards; two of the seven invited apprentices’ preceptors participated in FG‐1, while five of the nine invited preceptors of RNs and physiotherapy students (four RNs and one physiotherapist) participated in FG‐2. Thus, seven informants participated in the FGs, while two KIs gave individual interviews. Sick leave, vacations and not being able to leave the ward due to understaffing or lack of information were reasons given for not participating.

The two KIs represented resource persons who planned, arranged and facilitated the preceptor‐team meetings; the external advisor and one ward leader representing the NH management participated. These informants were considered to possess comprehensive information about the implementation of the preceptor‐team (Brinkmann & Kvale, 2015).

### 4.2 Data collection

The FG discussions and the KI interviews were conducted using semi‐structured interview guides. The participants’ experiences of being a preceptor, the participants’ experiences of attending the preceptor‐team and the organization of preceptorship at the wards were focused during the discussions and interviews. The first author (moderator) and the second author conducted the FGs, which were tape‐recorded. The second author completed the verbatim transcriptions. Due to bad sound recording, identifying the individuals in FG‐2 was impossible. Thus, the data reflected the group's interaction and communication and not the individuals. Table 1 lists background information collected at the beginning of the FG discussions.

**Table 1:** Participant characteristics

Age (years)	20–39	3
	40–46	5
Work experience (years)	1–5	2
	6–20	3
	≥21	3
Extensive education in precepting	Yes	4
	No	3
Times precepting apprentices or students	1–3	4
	4–6	3
Attended precepting seminars at college or university	Yes	3
	No	4
Times attending the precepting team meetings	1–3	2
	4–6	5

### 4.3 Analysis

A thematic analysis comprising five steps was conducted following Brinkmann and Kvale (2015, pp. 233–235). First, all authors read the transcriptions of the FG discussions and the KI interviews several times to grasp the overall meaning. The first author (BA) followed the next four steps. During several meetings, the research group commonly discussed and reflected. Second, the text was divided into meaning units, which were given a code and condensed as close as possible to how they were expressed by the informants. Third, the condensed summaries were interpreted for their underlying, latent meaning and BA developed an analytic text based on the summaries. Fourth, the analytic texts were scrutinized, evaluating their ability to answer the research question and themes and subthemes that represented patterns in the underlying, latent meaning were developed. Fifth, the non‐redundant themes were tied together into comprehensive, descriptive statements and quotes reflecting the content were assigned. Finally, to shed light on the data and to clarify and conceptualize the themes, theoretical perspectives were carefully applied. The leading question in search of an appropriate theoretical framework was “Why didn't the preceptors and the leaders do what they had agreed to do according to the implementation plan?” The second, third and fourth steps were conducted several times in an iterative process. The authors addressed the transcriptions several times to ensure that the empirical material underpinned the findings emphasized.

In this study, all authors were RNs, held an MSc and were widely experienced in teaching and supervising health professional students. In addition, two of the authors held a PhD. The first and last authors have previous experience as NH leaders in Norway. The third author has considerable experience as a leader of higher health professional education.

### 4.4 Ethical considerations

Participants received verbal and written information about the aim of the study, confidentiality of the data and their right to withdraw at any time without stating any reason. All participants provided informed, voluntarily written consent. The study was approved by the Norwegian Data Protection Official (NSD, ref. no. 38870/3/LB). No patients were involved, and no patient information was used in this project.

## 5 RESULTS

The findings displayed a main theme: “Precepting is a burden that someone must bear.” The subordinated themes represented that “Colleagues should not support precepting,” “Leaders should not facilitate precepting” and “The preceptor‐team is an arena for sharing frustration.”

### 5.1 Precepting is a burden that someone must bear

Precepting as a burden that someone must bear expressed the culture about preceptorship in the NH as an organization. Contributing to the education of health personnel was not considered an obligatory part of the job, but it still was an obligation targeting health professionals as individuals. To fulfil the obligations towards precepting students and apprentices, the preceptors carried an individual burden:

We are legally required to be preceptors. We have an occupation that requires clinical education in addition to theoretical knowledge. Moreover, we are practitioners ... so we need to have students to whom we can provide clinical guidance. (FG‐2)

The preceptors reported that in spite of feeling obliged to take on the task, they found precepting to be personally and professionally beneficial:

It [being a preceptor] implies of course that we must stay updated too. They [students] can often come and ask difficult questions… We get a little sharpened… Although we are required to be preceptors… and although it can be rather laborious at first, then it goes by and they become a resource at the ward. (FG‐2)

Precepting competency was considered individually, as not belonging to the ward and therefore not prioritized. Several informants did not gain possibilities to increase their competencies in precepting:

I think it is important to participate at these gatherings [at the school] and that the school should invite [practitioners]. What happens is that the leaders keep this information [the invitation] for themselves. It would have been important to work through the curricula, etc. (FG‐2)

Thus, the preceptors seemed familiar with the NH cultural interpretations of precepting as a burden the individual preceptors were supposed to bear on their own.

### 5.2 The clinical staff should not support precepting

Precepting and attending the team meetings were deemed activities outside the wards’ daily activities. Thus, the preceptors were mentally prepared to prioritize other activities at the ward and thus skip the precepting if needed:

You put your colleagues in trouble [when leaving for precepting or team attendance] – which is a bad feeling. (FG‐1)

The preceptors did not find this understanding inappropriate, but rather quite normal and understandable. Conversely, these experiences were also stressful as they lost time to fulfil their obligations of precepting the students. Similarly, attending the preceptor‐team also implied leaving the ward, which was considered a punishment of the colleagues; they had to “pay for” preceptors’ leaving. Even when attending the team, the preceptors’ minds were concerned about tasks at the ward, indicating that they were not present. Some preceptors left the team before the meeting ended to tend to other duties:

To leave early [go before agreed time from preceptor‐team] because there is not time enough… and the staffing [at the ward] is scarce. (FG‐2)

The preceptors of the physiotherapists, on the contrary, did not refer to precepting as a burden. This seemed related to the fact that their work community was less dependent on these preceptors in the daily work, providing better possibilities for adjusting their time for precepting:

It turns out to be more difficult to the nursing personnel to leave for precepting tasks… for us as the physiotherapists, leaving the clinical work for precepting does not become a strain on our colleagues as it does to the nurses … for us, it is not so hard to free time for preceptorship. (physiotherapist FG‐2)

The preceptors of the apprentices expressed more eagerness to engage both colleagues and leaders in discussions about precepting as well as demanding time to attend the preceptor‐team. However, they also felt less empowered to bring about change among their colleagues and to be noticed by their leaders:

No, we are not considered the most important occupational group.

When you speak out again and again [about allocating time for precepting at the ward’s schedule], you’re not getting popular. (Dialogue between participants FG‐1)

The culture for precepting punished those opposing it; challenging the cultural perceptions was so tiring and inconvenient that they surrendered.

### 5.3 Leaders should not facilitate precepting

The ward leaders appointed preceptors among their staff, trying to find someone motivated for the role. The leaders held that they had fulfilled their obligations about scheduling and thereby established precepting as a part of the daily activities:

We put it [preceptors’ tasks] on the schedule as a task for the actual employee, in line with the tasks related to medicaments, supply and the dirty utility room… The preceptors have made me aware of their plans ahead so I can put them on our schedule…so yes. (KI—ward leader)

It seemed that the leaders introduced no other incentives to accomplish their responsibility for precepting, apart from making it visible at the ward's daily time schedule. When asked if they offered any other incentives, they replied:

No – it is just that we have tried to set aside time for precepting. (KI—ward leader)

The leaders reported that the staff was hardly willing to adjust their work schedule to facilitate preceptor's attendance at the preceptor‐team meetings. Besides, they faced a poor economic leeway to allocate additional staff to release dedicated time for precepting. Nevertheless, the ward leaders did not emphasize economic issues in their meetings with the municipality management.

Nonetheless, some preceptors described breach of promises in providing dedicated time for precepting students as well as time for attending the preceptor‐team, leading to feelings of disappointment towards the leaders:

It’s an example from today – the person who should switch schedules – nothing has been done with it – it seems like they [the leaders] do not pay any attention to it – it is how it is – and when nothing is put in the system from the management, it turns out to be difficult for us. (FG‐2)

Others experienced humiliation when confronting the leader with the lack of follow‐up:

For instance, at the previous appointment for FG, the one we actually should have, then, I had the late shift and asked for the morning shift … it did not work … I was told [by the leader] that “you cannot be everywhere, you know.” (FG‐1)

The preceptors and the leaders accused each other of not accomplishing the decision to implement the intervention. The willingness to prioritize precepting and integrate it into the wards’ regular activities appeared to be minimal.

### 5.4 The preceptor‐team is an arena for sharing frustration

The preceptor‐team meetings are intended to be an arena for sharing experiences and mutual support (Kvam, 2015). Among others, the relationship between the preceptor and the student/apprentice was focused during the preceptor‐team meetings (Kvam, 2015); the participants appreciated this. However, the issue that engaged them the most was the lack of time for precepting:

We have talked much about the use of time. To follow up with an apprentice, having freed time for precepting is mandatory… For example, the quarterly assignments and such things, it is all about getting some time freed. (FG‐1)

The preceptor‐team helped them to cope with their feelings of not being able to fulfil the expectations, but this was not a hotbed for changing their behaviour. Instead, the team was accustomed to confirming the collective sense of lack of time, giving each other a feeling of not being alone in their understanding of the situation. The preceptors of the students admitted that they had not changed their behaviour or planned precepting, nevertheless, stating that such changes were needed:

We have heard about it [the decision], but not done anything with it. (Informant 1, FG‐2)

[Laughing in the group]

I have forgotten the team meeting twice; it was not on the schedule*.* (Informant 2, FG‐2)

Still, they did not expect the ward leader to take part in the planning of the team meetings:

No, we need to take action ourselves; the leaders cannot afford to remember everything on our behalf. We have to grab the case of prioritising time ourselves. (FG‐2)

In this way, both the preceptors and the leaders continued to act as usual, discussing what to do without doing anything about it.

Lack of time became legitimate grounds for not showing up. Although all sixteen preceptors had agreed to participate in the preceptor‐team, fewer than 10 joined at five of the seven planned team meetings (Kvam, 2015). The external advisor did not expect that frustration related to lack of time and limited resources should dominate the team meetings:

I have wondered a lot about how we should work, because one thing is to give information – but information is not given before it is understood. How do we work on that? (KI, External advisor)

The external advisor did not offer any solution for how to deal with the lack of vigour among leaders and preceptors to accomplish the decisions of generating precepting to be a positive and attractive role for the staff to hold.

## 6 DISCUSSION

The aim of the present study was to investigate possible hinders for implementing the intervention of interprofessional preceptor‐teams. Consequently, the focus of this study was on the negatives. A main theme: “Precepting is a burden that someone must bear” and three subthemes: “Colleagues should not support precepting”; “Leaders should not facilitate precepting”; and “The preceptor‐team is an arena for sharing frustration” were identified. Together, the theme and the subthemes characterize the institutional traits in the NH. While the first represents the general interpretation and guidance in the NH about preceptorship as a burden for preceptors, the second and the third traits represent colleagues and the NH management. Consequently, this study also revealed three central carriers (Scott, 2014) of institutional traits: preceptors, colleagues and managers.

The remainder of this article discusses the institutional traits, as well as the interaction between the institutional traits and the intervention, and finally, possible implications for leadership in NHs are addressed. The institutional traits will be discussed in the light of (a) the obligations about precepting (“have to”), (b) the collectively formed norms about precepting (“ought to”) and (c) the motivation for precepting (“want to”)*.* Table 2 presents the three carriers and the corresponding institutional traits.

**Table 2:** Institutionalized traits related to precepting

	Precepting is a burden that someone must bear		
	Preceptors	Colleagues	Managers
Institutional traits			
Regulative elements (have to do)	Obligated when appointed	Not obligated	Obligated to appoint preceptors
Normative elements (ought to do)	The preceptors ought to fill the whole preceptorship role themselves	Colleagues should not be involved in precepting	Leaders should not engage in precepting
Cultural–cognitive elements (want to do)	The preceptors want to find time for precepting; they do not want to put burdens on their colleagues.	Colleagues do not want to make precepting become a daily activity	Leaders do not want to prioritize precepting

The regulative trait (Table 2) is the NH's collective interpretation of the juridical regulations about precepting. As an institution, the NH interprets these regulations to mean that the leaders are obligated only to designate preceptors; leaders have no further obligations related to precepting. The leaders try to avoid forcing anyone to precept. Accordingly, they search for preceptors who are motivated and “want to” be preceptors. The preceptors seem to be the extended reach of the education into clinical practice, which they found personally interesting and developing. Therefore, they accepted the preconditions, thus making precepting a “private enterprise,” which they tried to merge with their ordinary tasks. On the other hand, “want to” only represented the preceptors’ individual wishes and not the ward or the NH as an organization. Consequently, despite their individual desires, the preceptors “ought to” comply with the expectations of perceiving precepting as a burden. Thus, the preceptors themselves, their colleagues and their leaders behaved in accordance with “precepting is a burden.” To involve the NH organization in precepting remained not an “ought to” nor a “want to,” but a “have to.” The NH's involvement in precepting on an organizational level was kept as less influential as possible. Accordingly, the leaders did not prioritize precepting or the education of preceptors, did not dedicate time for precepting and did not involve colleagues in precepting. The NH staff behaved in accordance with “precepting is a burden”; precepting was an activity, where they neither had to nor ought to join. Consequently, the colleagues rejected, resisted or hesitated to contribute in precepting. The preceptors acted in accordance, hesitating and holding back from asking for assistance or time for precepting. Thus, the leaders, the preceptors and their colleagues manifested and amplified “perception is a burden,” that is, they performed as carriers of the institutional traits. Interestingly, the analysis also revealed another aspect about norms: it is appropriate for the preceptors, the colleagues and the managers to complain about the burden of precepting imposed on the NH. They all “ought to” complain about precepting. The institutional traits involve preceptors of apprentices and nursing students. However, the preceptors of the physiotherapy students reported less of a problem with lack of time. This health professional group was less involved in daily activities at the ward and therefore less exposed to the institutional traits.

The present findings support previous research about preceptors that report lack of time and support to prepare and accomplish precepting (Forber et al., 2015; Trede et al., 2016; Trede, McEwen, Kenny, & O'Meara, 2014). Trede et al. (2016, p. 268) state that “preceptors are primarily practitioners and only secondarily educators” (p. 268). The institutional traits found in this study reflect this statement. The collective recourses in the NH organization are primarily dedicated to clinical work, not educational work. The institutional traits have arisen from, are embedded in and preserve this situation. Previous research that primarily has investigated precepting from the educational perspective has noticed the preceptors’ workload as a problem but has not scrutinized this problem from an organizational perspective. This study adds new knowledge about how institutions perpetuate this situation by forming an internal logic of institutional traits that give meaning for the carriers of these traits, but that does not appear to be rational from an outsider's perspective. It is surprising that the leaders seemed to be scarcely concerned about the preceptors’ workload and working health, adding preceptorship to their ordinary task load while hardly providing any recourse or support at all. Trede et al. (2016) state that the support and training programme for preceptors, provided by the preceptor‐team in this study, might be contraindicated because such interventions might increase the risk for preceptor burnout unless organizational support is provided.

The institutional traits found (Table 2) might be specific to the featured NH. On the other hand, similar organizations like NHs tend to form organizational fields that share similar institutional traits because they tend to copy each other's “want, ought and have to” (Czarniawska & Joerges, 1996; Meyer & Rowan, 1977). A Norwegian study (Kårstein & Caspersen, 2014) demonstrated a general lack of organizational support when facilitating clinical learning for students. Omansky (2010) stated that despite intrinsic rewards, the preceptors experience a role ambiguity, conflict and overload when precepting students. Trede et al. (2016) reported that preceptors’ lack of additional hours dedicated to the role, lack of preparedness, derision by colleagues and loneliness in the role are major organizational problems that need to be sorted out. Rodger et al. (2008) reported failing prioritization of time and recourses of preceptorship for the international allied health professions.

The present intervention framed as the “interprofessional preceptor‐team” was intended to support preceptors and enhance their thriving and competencies in their role. However, the outcome turned out to be the opposite; attending the preceptor‐team became an additional burden including an individual dilemma of balancing personal interests of being present with obeying expectations to make precepting invisible to the collective. The preceptor‐team turned into an arena for complaining about lack of recourses for preceptorship and in this way amplified a collectively objection of precepting. In spite of an implementation plan that seemed to be logical and rational, the implementation in this case caused an unexpected result; the intervention added burdens to the preceptors’ role.

A lesson learned is that the instrumental approach to change is needed but is insufficient to achieve sustainable change. The latter requires all parties to address the institutional traits of the organization, that is, the collective interpretations of what has to be done, what ought to be done and what we want to be done (Palthe, 2014; Thelen & Mahoney, 2010). Nevertheless, processes of institutional change might also be incremental (Dacin, Goodstein, & Scott, 2002). From an institutional perspective, change occurs when some of those possessing organizational traits stop defending them, try to oppose them or introduce new ideas (Thelen & Mahoney, 2010). The present study revealed some traces of change: some preceptors, especially the preceptors of the apprentices, became aware of the need to claim available time for precepting. At the same time, as they considered themselves to be at the bottom of the hierarchy among the health professionals at the ward, they did not feel empowered to oppose the institutional traits, especially to the face of their colleagues to whom they are highly dependent in their work. The RNs seemed to have larger possibilities of self‐management and thus, to a lesser degree, opposed the institutional traits. Rather than opposing the traits, however, the RNs maintained them by explaining their rationale: In the RN's eyes, the institutional traits represented a fair reaction towards an unfear demand inflicted upon not only them as individuals, but also the NH as an organization. On the other side, both the nurse and physiotherapist preceptors addressed the leadership's responsibility to counteract the unfairness imposed to them as a collective. However, they did not confront the management with their thoughts. The preceptors perceived the resistance to change as so insurmountable that they chose not to confront either their leaders or their colleagues.

The distinction between the NH institutional traits and the individual thoughts is noteworthy. According to institutional theory, individuals tend to adjust their statements and behaviours in accordance with what they perceive as collectively acceptable. The forces related to institutional traits affect individuals’ behaviour: the stronger the forces, the less deviance from expected behaviour (Scott, 2014). While out of reach of the forces embedded in the institutional traits, individuals might feel free to express alternative thoughts and ideas. Individuals change more easily than institutions (Scott, 2014; Thelen & Mahoney, 2010). Thus, neither the frustrated, external change agents nor the preceptors and the leaders complied with their own decision to change. However, the responsibility for this failure to change should not be addressed to the preceptors and leaders as individuals. Instead, it should be directed to the institutional traits maintained and amplified by the interaction between all the carriers of these traits, that is, the preceptors, the colleagues and the leaders.

Managers have a responsibility to lead overall organizational change (Lewis, 2011; Reay et al., 2013; Thelen & Mahoney, 2010). Conversely, this study revealed that the management reinforced institutional traits. The managers formally established the project but seemed not to partake in the implementation of the interprofessional preceptor‐teams themselves. Instead, they delegated the change process to the external advisor and the preceptors, keeping precepting apart from the ordinary tasks at the ward and out of their responsibility as leaders. The present analysis revealed that even though the individual's personal opinion might be congruent with the new practice implemented, the institutional traits exerted strong pressure on the individual, causing him or her to act in accordance with the prevailing practice. Thus, aiming to change the individuals—in this case, the preceptors—might not lead to sustainable implementation (Proctor et al., 2011). This study indicates that implementation plans in NHs should include explicit steps for dealing with institutional traits (Thelen & Mahoney, 2010). Reay et al. (2013) found that managers’ engagement with clinical staff was crucial for collective meaning‐making and the institutionalization of new practices. This implies that the management needs to actively partake in the reinterpretation of what NHs have to do, ought to do and want to do about precepting of students and apprentices. The managers need to create arenas for collective meaning‐making and the more, to be present at these arenas supporting alternative interpretations of the NH's obligations about precepting. In doing so, the management can be aware of the traits, including their own contribution to the resistance to change. The present implementation plan probably failed due to ignoring the general need for arenas of collective meaning‐making processes and particularly the management's involvement in such processes. The preceptor‐team, the only arena for social interaction arranged in this project, was designed to target the preceptors’ individual needs of support and was therefore not intended to be an arena for elaborating institutional traits.

What might be the logic behind the traits revealed in this study? Forber et al. (2015, p. 1115) claimed that “legacies of the past” permeate the entire institutional field of healthcare services when it comes to precepting. The shift of health personnel education from hospital‐based apprentice training to degree‐level preparation in the 1970s led to an uncoupling of health and education and a leadership vacuum (Forber et al., 2015). The formal organizational responsibility for the education of health professionals was moved from the healthcare services to the universities, while studies were still preserved at clinical sites as needed to achieve learning outcomes (Forber et al., 2015). In the light of this background, the institutional traits expecting precepting to be almost invisible among the daily activities in the healthcare services might be viewed as logical and rational. On the other hand, the institutional traits revealed in this study counteract the interests of the healthcare services as well as those of the universities and colleges when it comes to both recruitment and retention of preceptors.

Some limitations of this study should be noted. Among 16 preceptor‐team members invited, only seven participated in the FGs, forming two FGs representing four wards. Dropouts among the invited informants might likely have decreased the quality of the findings by limiting the variety of experiences and thereby the rigour of the study. FG‐1 comprised only two out of seven invited informants, making a very small group. Sick leave, vacations and not being able to leave the ward due to understaffing and lack of information were reasons given for not attending the FGs. The presence of more informants would probably have provided more nuance, depth and width to the themes at stake. Nevertheless, FG‐1 created a reflective, nuanced, engaged and interesting dialogue, built on each other's statements. Towards this end, this FG met the intentions. The present sample included preceptors with both extended as well as scarce experience in preceptorship. Thus, the participants' varied experiences are a strength of this pilot study. However, one NH signifies a limited database for transferability of the findings.

## 7 CONCLUSIONS AND IMPLICATIONS

Institutional traits may hamper interventions to improve the working conditions of preceptors for students and apprentices in their clinical studies in NHs. The institutional traits, including the perceptions held by preceptors, leaders and colleagues of the rule‐like understanding of the “want to,” “ought to” and “have to,” preserved precepting as apart from normal activities in the NH. This situation frustrated the preceptors, while attempts from actors outside the NH to improve the conditions for preceptors seemed to be counteractive as they added burdens to the preceptors’ role. Thus, one should consider whether interventions to increase preceptors’ competencies could be a waste of resources and even harmful if the working conditions of the preceptors are not considered.

This study adds knowledge about how management in NHs might maintain work conditions that cause major stress and burdens for preceptors of health professional students and nursing associate apprentices. NH leaders should actively partake in dialogues with the educational sites to clarify their role and responsibilities about preceptorship for students and apprentices. Implementation plans that do not address institutional traits should be rejected. Institutional traits are carried by individuals, but operate collectively as cultural influences, which might cause resistance to change. More research is needed to investigate how institutional traits interact with intentions to change organizational behavioural in NHs about preceptorship of students and apprentices.

## CONFLICT OF INTERESTS

We declare no conflict of interests.

## AUTHOR CONTRIBUTIONS

BA and GH: Study design. BA (moderator) and VS: Focus group conduction. BA: Drafting of the manuscript. All authors analysed the data, contributed to editing the final manuscript and then all revised it critically for scientific content, read and approved the final version.

All authors have agreed on the final version and meet at least one of the following criteria [recommended by the ICMJE (https://www.icmje.org/recommendations/)]:

- substantial contributions to conception and design, acquisition of data or analysis and interpretation of data;
- drafting the article or revising it critically for important intellectual content.
